# Multi-Modal Glioblastoma Segmentation: Man versus Machine

**DOI:** 10.1371/journal.pone.0096873

**Published:** 2014-05-07

**Authors:** Nicole Porz, Stefan Bauer, Alessia Pica, Philippe Schucht, Jürgen Beck, Rajeev Kumar Verma, Johannes Slotboom, Mauricio Reyes, Roland Wiest

**Affiliations:** 1 Support Center for Advanced Neuroimaging - Institute for Diagnostic and Interventional Neuroradiology, University Hospital Inselspital and University of Bern, Bern, Switzerland; 2 Department of Neurosurgery, University Hospital Inselspital and University of Bern, Bern, Switzerland; 3 Institute of Surgical Technology and Biomechanics, University of Bern, Bern, Switzerland; 4 Department of Radiation Oncology, University Hospital Inselspital and University of Bern, Bern, Switzerland; University of Iowa, United States of America

## Abstract

**Background and Purpose:**

Reproducible segmentation of brain tumors on magnetic resonance images is an important clinical need. This study was designed to evaluate the reliability of a novel *fully automated* segmentation tool for brain tumor image analysis in comparison to manually defined tumor segmentations.

**Methods:**

We prospectively evaluated preoperative MR Images from 25 glioblastoma patients. Two independent expert raters performed manual segmentations. Automatic segmentations were performed using the ***Bra***in ***Tum***or ***I***mage ***A***nalysis software (BraTumIA). In order to study the different tumor compartments, the complete tumor volume TV (enhancing part plus non-enhancing part plus necrotic core of the tumor), the TV+ (TV plus edema) and the contrast enhancing tumor volume CETV were identified. We quantified the overlap between manual and automated segmentation by calculation of diameter measurements as well as the Dice coefficients, the positive predictive values, sensitivity, relative volume error and absolute volume error.

**Results:**

Comparison of automated versus manual extraction of 2-dimensional diameter measurements showed no significant difference (p = 0.29). Comparison of automated versus manual segmentation of volumetric segmentations showed significant differences for TV+ and TV (p<0.05) but no significant differences for CETV (p>0.05) with regard to the Dice overlap coefficients. Spearman's rank correlation coefficients (ρ) of TV+, TV and CETV showed highly significant correlations between automatic and manual segmentations. Tumor localization did not influence the accuracy of segmentation.

**Conclusions:**

In summary, we demonstrated that BraTumIA supports radiologists and clinicians by providing accurate measures of cross-sectional diameter-based tumor extensions. The automated volume measurements were comparable to manual tumor delineation for CETV tumor volumes, and outperformed inter-rater variability for overlap and sensitivity.

## Introduction

Glial tumors are the most frequent primary brain tumors in adults, accounting for 70% of adult primary cerebral malignancies. Glioblastoma (GBM), the most common malignant primary brain tumor in humans, exhibits very rapid infiltrative growth and a poor prognosis, with an average survival time after GBM diagnosis of one year [1]. In recent years, the overall survival of glioblastoma patients has increased due to more extensive treatment strategies such as concomitant radio- and chemotherapy [2] and advanced surgical techniques [3] like fluorescence-guided surgery with 5-aminolevulinic acid (5-Ala) or advanced mapping methods [4], [5].

Prognostic biomarkers for improved survival include imaging of the extent of resection based on the amount of postoperatively enhancing tissue as determined by structural MRI. Depending on the study populations, gross resection of 78% to 98% of the enhancing tumor volume is associated with an improved survival in patients with previously untreated or recurrent GBM [6], [7]. Preoperative imaging characteristics associated with better survival are: i) absence of necrosis, ii) amount of contrast enhancing tumor, and iii) extent of solid tumor exceeding the enhancement [8], [9].

Whole tumor and sub-compartment segmentation is performed manually in most centers and is still considered the “gold standard” procedure. Several studies reported failures of individual expert rater segmentations because structural MRI may obscure the precise visual delineation of glioma boundaries [10]–[12]. In addition, manual segmentation is time-consuming and is a rate-determining step for further treatment planning [13]. Fully automated user-independent segmentation tools are available for research purposes. However, up to now they were considered limited with regard to ease of use, accuracy and speed, and therefore not adequate for clinical applications [14].

There is a large body of literature available on automatic brain tumor segmentation [14], but only recently the focus is shifting towards segmentation of individual tumor sub-compartments from multi-modal images. Most of the relevant fully automatic brain tumor segmentation methods employ a supervised or unsupervised tissue classification, that assigns labels based on voxel-wise or regionally extracted features. Corso et al. [15] segmented tumor core and edema using Bayesian tissue classification fused with affinity assignment by weighted aggregation. Verma et al. [16] used a support vector machine classifier to segment necrotic, active and edema tumor tissues. Bauer et al. [17] followed a similar approach, but added regularization constraints to increase robustness. Menze et al. [18] combined an atlas of normal individuals with a latent tumor atlas to employ a generative model for segmenting tumor compartments in different modalities. Zikic et al. [19] used context-aware voxel features as input for a decision forest classifier to segment 3 different tumor compartments from multi-modal images. A comparison of the performance of many recent methods on a standardized research dataset can be found in [20].

Almost all evaluations so far focus on the assessment of overlap measures like Dice coefficient, which are well established for comparing segmentation algorithms. However, other clinically more relevant measures like the diameter-based RANO metrics [21], volume error or the tendency to over- or undersegment are hardly ever reported. Moreover, there are only very few methods, for which the source code is publicly available (e.g. [22], [23]), and to the best of our knowledge there exists not a single publicly available fully automatic segmentation tool with a graphical user interface that can be used by non-specialists and that can be applied to clinical images directly.

This study was designed to compare a novel clinically-oriented *fully automated* segmentation tool for brain tumor image analysis with manually defined tumor segmentations by two independent raters. Specifically, we aimed to evaluate: i) whether the fully automated tool can reproduce current 2D diameter-based criteria for brain tumor assessment [21]; ii) whether volumetric criteria can be reliably estimated by fully automated segmentation, including susceptibility to failure within dedicated sub-compartments. Furthermore, we also report iii) whether tumor localization has an effect on the quality of automated segmentations; iv) how improved image acquisition protocols impact the segmentation result.

## Methods

### Study population

Patients with newly diagnosed and histologically confirmed glioblastoma pre-operatively admitted to our institution between October 2012 and July 2013 were eligible for this prospective study. Exclusion criteria were: incomplete image acquisition, Karnofsky performance status <70%, abnormal hematologic, renal or hepatic function, and previous cranial neurosurgery. The study was approved by the Local Research Ethics Commission (Kantonale Ethikkommission Bern). All patients provided written informed consent.

### MR Imaging Protocol

MR images were acquired on two different 1.5 TMR scanners (Siemens Avanto and Siemens Aera, Siemens, Erlangen/Germany). Every patient underwent a standardized MRI protocol including: i) 3D-T1w-MPR in sagittal acquisition, 1 mm isotropic resolution; ii) post-contrast 3D-T1w-MPR in sagittal acquisition, 1 mm isotropic resolution; iii) 3-D T2w (SPC) in sagittal acquisition, 1 mm isotropic resolution; iv) fluid-attenuated inversion recovery (FLAIR) (TIR 2D) in axial acquisition. The sequence parameters were as follows: for pre-contrast 3D-T1w-MPR sequences TE = 2.67 ms, TR = 1580 ms, FOV = 256×256 mm^2^, FA = 8°, with an isotropic voxel resolution of 1 mm×1 mm×1 mm; for post-contrast T1w TE = 4.57 ms, TR = 2070 ms, FOV = 256×256 mm^2^, FA = 15°, using isotropic 1 mm×1 mm×1 mm voxels; for 3D T2w (SPC) in sagittal acquisition TE = 380 ms, TR = 3000 ms, FOV = 256×256 mm^2^, FA = 120°, using isotropic 1 mm×1 mm×1 mm voxels; for the 2D fluid-attenuated inversion recovery sequence TE = 80 ms, TR = 8000 ms, FOV = 256×256 mm^2^, FA = 120°, using a non-isotropic voxel size 1 mm×1 mm×3 mm.

### Re-evaluation of pooled datasets from the MICCAI BraTS challenge

In order to compare the impact of the modified imaging protocols on the segmentation quality, we additionally analyzed a dataset composed of MR images from the MICCAI 2012 Brain Tumor Segmentation (BraTS) challenge consisting of in-silico and research datasets of brain tumor patients [20]. This dataset is different from the dataset in our clinical study described above. The BraTS images included the same MRI sequences with lower resolutions and various parameter settings. Quality of the automated segmentation was tested compared to the manually generated ground truth using the online evaluation tool (http://www2.imm.dtu.dk/projects/BRATS2012).

### Two-dimensional measurements

In order to compare the accuracy of automated versus manual segmentation, we computed the product of two maximum diameters for 2-dimensional measurements, as recommended for reporting of gross tumor volume by the WHO and the refined RANO guidelines [21], [24]. The automated 2-dimensional measurements were extracted from the largest perpendicular diameters of the contrast-enhancing lesions of the automatically segmented tumor expansion. These were compared to the measurements of our two expert raters using the product of maximal cross-sectional enhancing diameters. In case of multiple lesions, the sum of products of diameters (SPD) of all lesions was calculated [25].

### Manual volumetric segmentation

The manual segmentations were performed by two independent expert raters: an expert in brain tumor imaging (SB) and a neurosurgeon experienced in brain tumor analysis (NP). Both raters were supervised independently by two neuroradiologists with more than 10 years of experience in brain tumor imaging (RW and RKV). Manual segmentation was performed with the open source software 3D Slicer Version 4.2.2.3 (www.slicer.org) [26]. Every patient (n = 25) was segmented manually slice by slice. Segmentation was performed on T1w, T1wGd, T2w and FLAIR sequences according to the Vasari MR feature guide v.1.1 (https://wiki.nci.nih.gov/display/CIP/VASARI). Four different tumor compartments were classified: i) non-enhancing tumor, ii) enhancing tumor, iii) necrosis and iv) edema. Hence, non-enhancing tumor was defined on FLAIR, T2w and T1w. Enhancing tumor parts were classified on post-contrast T1 weighted images compared to pre-contrast T1-weighted images excluding hemorrhage. Necrosis was defined as a region within the tumor that did not enhance and had a hyper-intense signal on T2w and FLAIR. Edema was classified on FLAIR, T1w, T2w and contrast-enhanced T1w [27]. The average time for manually segmenting all subcomponents of one study patient was approximately one hour.

### Automated Segmentation

Automatic segmentations were performed using the ***Bra***in ***Tum***or ***I***mage ***A***nalysis (BraTumIA) software (available under this link http://www.istb.unibe.ch/content/research/medical_image_analysis/software/index_eng.html). The software offers a completely integrated segmentation pipeline, where the user only has to load the original Dicom stacks of the four relevant MRI modalities (T1w, contrast-enhanced T1w, T2w, FLAIR). Then, the images are processed in a fully automatic way, including skull stripping [28] and subsequent rigid co-registration [29] to ensure voxel-to-voxel correspondence between the different MRI sequences. Based on the registered images, segmentation into unaffected tissue and tumor tissue, encompassing four different sub-compartments, is performed based on combined supervised classification and regularization. The algorithmic core of the segmentation evolved out of [17] and the basics of the current approach have been recently described in a conference paper [30]. Briefly, the segmentation problem is formulated as an energy minimization task in a conditional random-field context. From each voxel, a high-dimensional feature vector is extracted, consisting of multi-modal intensities, texture and gradient statistics from local image patches, multi-scale symmetry features across the mid-sagittal plane and location features. Based on this high-dimensional feature vector, each voxel is assigned a tissue label by a decision forest classifier [31]. Spatial constraints and prior knowledge are considered by a conditional random field regularization to increase robustness. More details are given in Data S1 and in [30].

**Figure 1:**
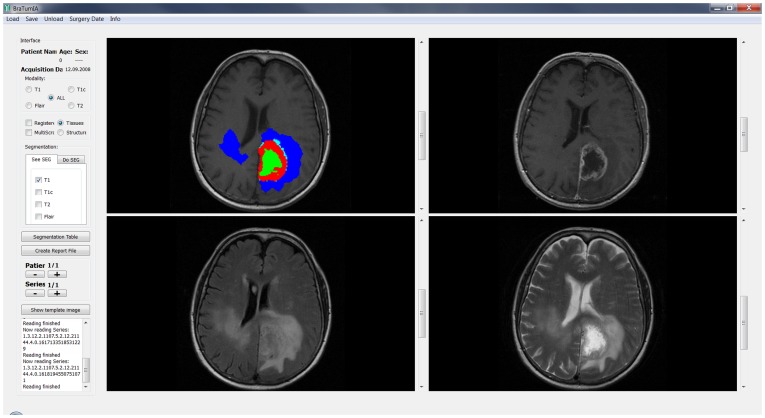
Graphical user interface of the BraTumIA software. Data can be loaded from the buttons at the top, the left side offers different options for processing and visualization and the largest part of the screen depicts the different MRI modalities with optional overlay of the segmentation results.

The segmentation method requires training on manually segmented images of brain tumor patients. Training was performed on 36 separate patients that were not part of the study. Computation time for the complete automatic processing was less than 5 minutes. The algorithmic core of the software was among the best performing methods at the MICCAI Brain Tumor Segmentation (BRATS) challenge (http://www2.imm.dtu.dk/projects/BRATS2012/). A screenshot of the user interface of the integrated software is shown in figure 1.

### Statistical methods

We evaluated the results primarily using the Dice coefficient [32]. The Dice coefficient measures the overlap of two regions (i.e. region 1 given by a manual segmentation, region 2 given by the automatic segmentation). It can range from 0 to 1, with 0 indicating no overlap and 1 indicating complete overlap. Additionally, we also report the positive predictive value (PPV) [33], sensitivity [34] and volume error between manual and automatic segmentations. The Wilcoxon signed rank test [35] was used for the statistical analysis of the difference between manual and automated volumetric segmentation in terms of the metrics mentioned above. For inter-observer comparisons and automatic versus manual comparisons, we also used Spearman's rank correlation coefficients [36]. For the comparison of 2-dimensional measures and investigation of the impact of localization, non-parametric analysis of variance [37], [38] was performed. Statistical analysis was done using Graphpad Prism version 5.

## Results

### Study population

The mean patient age at pre-operative MR imaging (+/− standard deviation (SD)) was 67.75 years +/−6.191 (range 53–79 years), mean pre-operative Karnofsky performance status (+/−SD) was 84.38% +/−6.292 (range 70–90%), and mean pre-operative NIHSS (+/−SD) was 0.25+/−1.12 (range 0–3). Of the 25 patients, 10 were female and 15 male. Five patients underwent stereotactic biopsy, 11 subtotal extirpations and nine complete resections of enhancing tumor (CRET). All diagnoses were confirmed by histopathology.

### Comparison of automated versus manual segmentation

#### Two-Dimensional measurements

**Figure 2:**
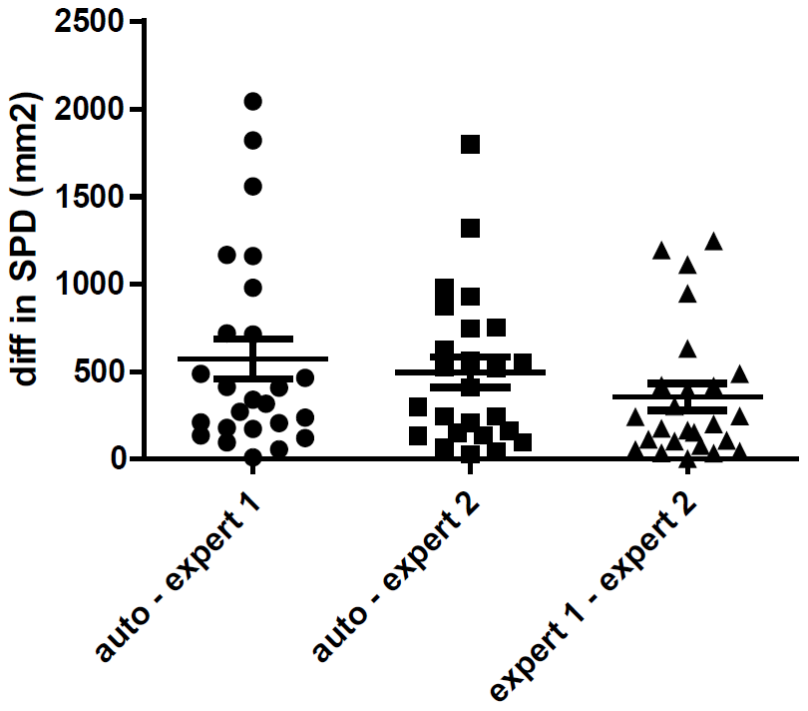
Comparison of the difference in 2- dimensional measurements of two expert raters and the diameters extracted from the automatic segmentations on the study population of 25 patients. SPD =  sum of products of diameters in mm^2^. Wide horizontal bars indicate the mean and the shorter horizontal bars indicate the SD.

Comparison between automated and manual 2-dimensional measurements by two expert raters was done using the product of maximal cross-sectional enhancing diameters (SPD in mm^2^+/−SD). The mean absolute difference in SPD of automatic diameter versus expert rater 1 was 570 mm^2^ (+/−569), (95% CI [335, 805]); the mean absolute difference in SPD of automatic diameter versus expert rater 2 was 495 mm^2^ (+/−438), (95% CI [314, 676]). The mean absolute difference in SPD of expert rater 1 versus expert rater (+/−SD) was 355 mm^2^ (+/−380), (95% CI [198, 511]). There was no significant difference between the three different measurements (Friedman test; p = 0.29). The results are depicted in figure 2.

#### Volumetric Segmentation

**Figure 3:**
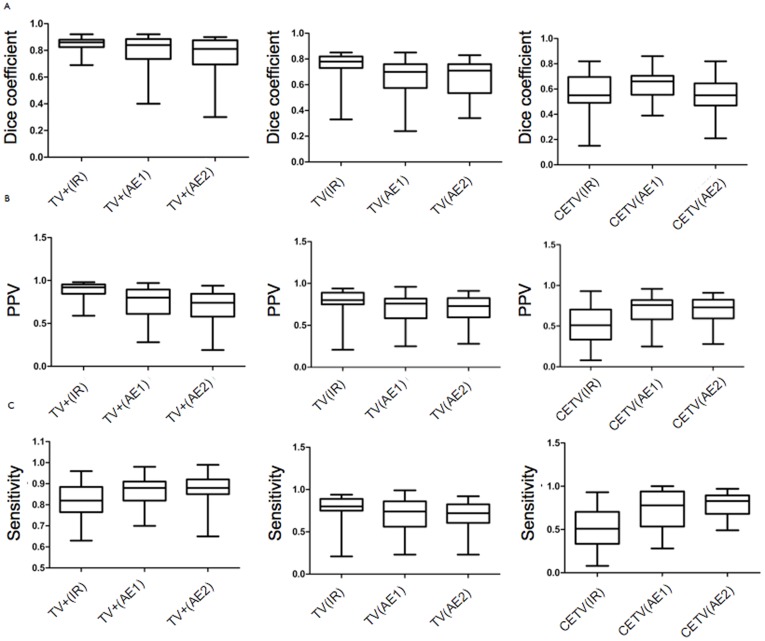
Box and whisker plots (min -max) of TV+, TV and CETV between manual raters (inter-rater (IR)), automatic segmentation versus expert rater 1 (AE 1); and automatic segmentation versus expert rater 2 (AE 2) for A: Dice coefficients B: PPV =  Positive predictive values C: Sensitivity for the sub-compartment segmentations.

**Figure 4:**
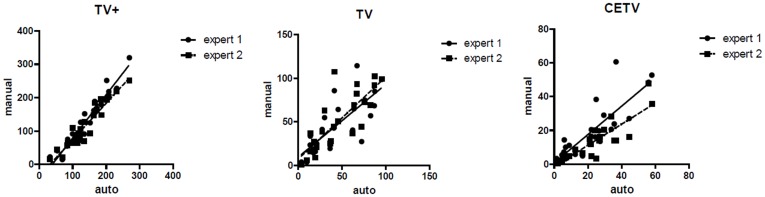
Scatter plot of the absolute volume measurement for TV+, TV and CETV (from left to right). Volumes measured automatically are shown on the x-axis, volumes measured manually by the two expert raters are shown on the y-axis.

To evaluate the different tumor compartments, we separately investigated the complete tumor volume (TV) encompassing the enhancing part of the tumor, the non-enhancing part of the tumor plus the necrotic core, the TV+ (TV plus edema) and the contrast enhancing tumor volume (CETV) of the GBM. We quantified the overlap between manual and automated segmentation by calculation of the Dice coefficients, the positive predictive values (PPV), sensitivity, relative volume error and absolute volume error. The results are summarized graphically with boxplots in figure 3. Average Dice coefficients for the automatic segmentation were 0.8 for TV+, 0.66 for TV and 0.63 for CETV (compared to expert 1 who defined the ground truth, see table 1). The average absolute volume error of the automatic segmentation was 20.4 ml for TV+, 14.5 ml for TV and 7.2 ml for CETV (compared to expert 1 who defined the ground truth). The relationship between automatically and manually calculated volumes of individual tumor sub-compartments is shown with scatterplots in figure 4. Details can be found in Data S1.

**Table 1:** Statistics of Dice coefficients from the MICCAI BraTS 2012 testing dataset compared to the Dice coefficients on our clinical study dataset (CS).

Dice	TV+	TV	CETV
BraTS median	0. 69	0. 55	0. 58
BraTS mean	0. 71	0. 55	0. 54
BraTS SD	0. 15	0. 19	0. 24
			
CS median	0.84	0.70	0.66
CS mean	0.80	0.66	0.63
CS SD	0.12	0.14	0.12
			
p-value	0.06	0.06	0.01

Using the Wilcoxon signed ranks test, we observed significant differences for TV+ and TV (p<0.05) but no significant differences for CETV (p>0.05) with regard to the Dice coefficients. Spearman's rank correlation coefficients (ρ) of the Dice coefficient for TV+, TV and CETV showed highly significant correlations (p-value) between automatic and manual segmentations (see Table 2).

**Table 2:** Spearman's rank correlation coefficients (ρ) of TV+, TV and CETV.

	AE1 versus AE2		AE1 versus IR		AE2 versus IR	
Dice coefficient	ρ	p-value	ρ	p	ρ	p
TV+	0.85	0.00	0.74	0.00	0.79	0.00
TV	0.90	0.00	0.68	0.00	0.75	0.00
CETV	0.42	0.03	0.51	0.00	0.84	0.00

**Figure 5:**
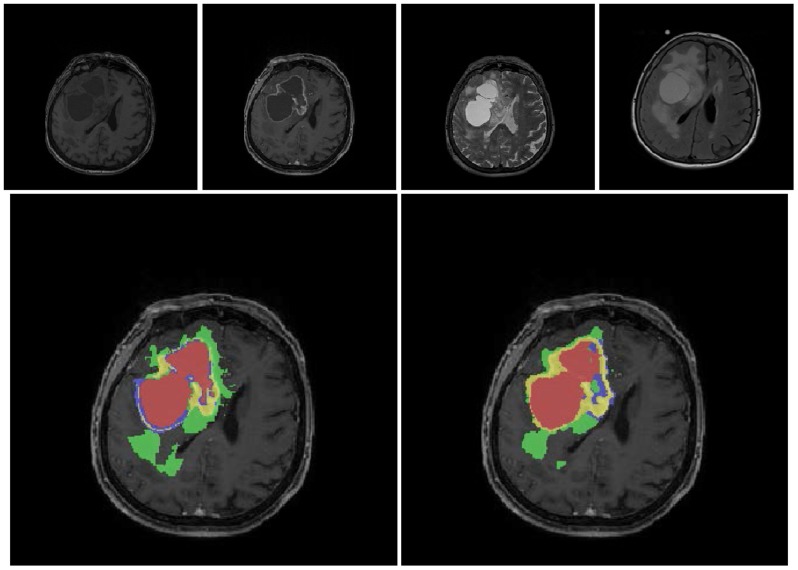
The figures show the original images and the segmentations as overlays on the post-contrast T1-weighted images for a patient with a good overlap of manual and automatic segmentation. Upper row: an axial slice of the original images (T1w, T1wGd, T2, FLAIR from left to right). Bottom row left column: manual segmentation, right column: automatic segmentation. Color code for segmentations: red  =  necrosis, yellow  =  enhancing tumor, blue  =  non-enhancing tumor, green  =  edema. TV+ corresponds to a combination of all colors, TV corresponds to red+yellow+blue compartments, CETV corresponds to the yellow compartment.

**Figure 6:**
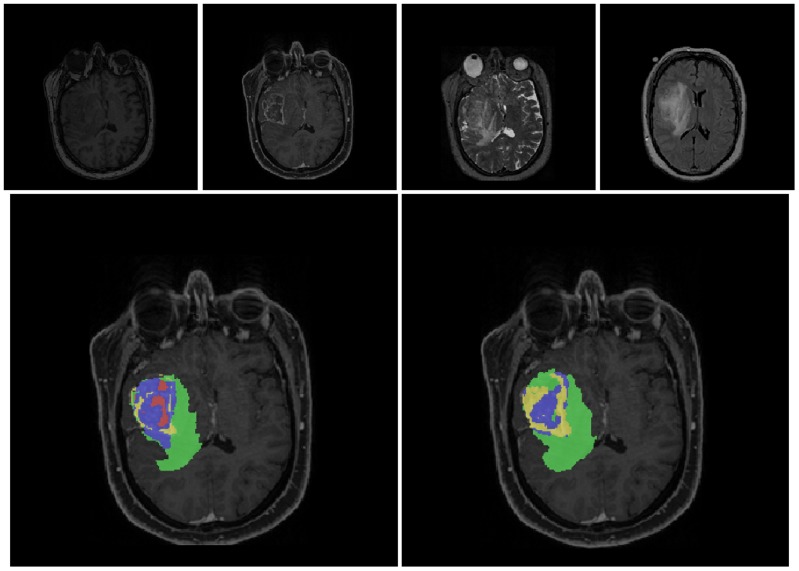
The figures show the original images and the segmentations as overlays on the post-contrast T1-weighted images for a patient with a moderate overlap of manual and automatic segmentation (CETV does not match well). Upper row: an axial slice of the original images (T1w, T1wGd, T2w, FLAIR from left to right). Bottom row left column: manual segmentation, right column: automatic segmentation. Color code for segmentations: red  =  necrosis, yellow  =  enhancing tumor, blue  =  non-enhancing tumor, green  =  edema. TV+ corresponds to a combination of all colors, TV corresponds to red+yellow+blue compartments, CETV corresponds to the yellow compartment.

Figure 5 illustrates results for a patient where manual and automatic segmentation match well, whereas figure 6 depicts a patient where only moderate agreement between manual and automatic segmentation could be achieved, mostly due to a mismatch of the CETV compartment.

#### Tumor localization and impact on accuracy of segmentation

Of the 25 analyzed data sets, 8 tumors were in the frontal lobes, 8 temporal, 8 parietal and one occipital (the latter was excluded from group analyses). In order to identify a potential impact of the tumor localization in different lobes of the brain on the accuracy of automated segmentation, we analyzed the influence of the tumor localization (frontal, temporal, parietal) on the Dice overlaps. A Kruskal-Wallis test revealed no differences related to the localization or the sub-compartments (see Table 3).

**Table 3:** Regional Differences in Dice overlap according to localization.

Dice	Frontal	Temporal	Parietal	p-Values
**TV**+ mean	0. 84	0. 81	0. 85	0. 62
**TV** mean	0. 65	0. 64	0. 74	0. 13
**CETV** mean	0. 57	0. 65	0. 68	0. 2

#### Segmentation results on non-standardized imaging protocols

Comparison of the Dice coefficients for automatic segmentation between the non-standardized MICCAI BraTS 2012 test data and the standardized high resolution datasets in our study patients revealed improved Dice coefficients for the standardized protocol in all sub-compartments (see Table 1). However the improvement was not statistically significant in all compartments (p = 0.06) due to the small number of patients in the BraTS dataset (11 patients).

## Discussion

In this prospective study we validated an automatic multimodal segmentation software (BraTumIA) in clinical practice. We delineated different tumor compartments, i.e. TV, TV+ and CETV in de novo GBM. Our goal was to evaluate how BraTumIA performed in our clinical setting in comparison to manual ratings. We focused on two metrics for clinical evaluation: the SPD according to the WHO classification [24], [39], and the volumes and overlaps of TV, TV+ and CETV. It was shown that automatic volumetry offers significant time gains compared to manual volumetry.

Two points stand out among the study results: i) computation of cross sectional SPD using automated 2-dimensional tumor extensions is comparable to manual tumor delineation; ii) estimation of TV, TV+ and CETV by the automatic method reaches a sensitivity, which is comparable to the inter-observer variability between two experts performing manual segmentation. For CETV, BraTumIA had a better overlap with the ground-truth than Dice overlap between manual inter-rater segmentations.

Furthermore, we observed that classification of automated segmentation is insensitive to tumor localization and individual compositions of the sub-segments of GBM. Comparison of the quality of the automatic segmentations on our new prospective patient dataset of standardized images with the quality obtained on the non-standardized BraTS 2012 images, showed an average improvement of more than 10% (table 1). This indicates that a standardized high-resolution isotropic imaging protocol such as the one used in this study has the potential to optimize the quality of automatic segmentations.

A previous study demonstrated that an extent of resection (EOR) of the TV ≥ 78% impacts patient outcome, and that the positive relationship between EOR and patient outcome can be observed even at the highest levels of resection [8]. For the pre-operative baseline assessment, we observed good concordance of linear SPD assessment between manual raters and the automated approach, and a higher sensitivity for automated volumetry of the CETV compared to two manual raters (72% vs. 53%, figure 3). Although the linear SPD measures provided a good concordance between automatic and manual measurements, these measures are problematic in cases of non-solid GBMs [40]. GBMs usually appear with a complex morphology, thus necessitating volumetric analyses. However, diameter-based SPD measures only consider one tumor compartment (necrosis+enhancing tumor), usually in low resolution datasets.

Most segmentation techniques focus on reporting of Dice similarity coefficients of the gross tumor volume instead of individual tumor sub-compartments. A semi-automatic approach using Slicer recently reported an average Dice similarity coefficient of 0.80 [41]. Semi-automatic segmentations tend to produce better accuracies compared to fully automatic methods but previous studies suggest that semi-automatic methods are less objective [42]. While previous approaches aimed to assess only one tumor compartment, we analyzed different metrics for TV+, TV and CETV individually. BraTumIA extends our understanding of complex morphology because it includes the edematous and necrotic parts of the tumor. The analysis is more challenging if several tumor regions are considered separately, but this differentiation offers a window for improved integration of other modalities such as diffusion or perfusion parameters [43]. In addition to pre-operative assessment, knowledge about volume changes beyond the CETV is important for the management of tumor recurrence and response to chemo- and anti-angiogenic therapy, since CETV are prone to errors due to pseudo-response and pseudo-progression [44]. Objective estimates of CETV after gross resection of the TV may improve risk assessment of tumor recurrence if the pre-treatment TV and CETV are precisely determined. This sub-compartmental approach may also provide a basis for the integration of further prognostic markers to differentiate pseudo-response or pseudo-progression and may therefore be of crucial significance for clinical use.

Accurate segmentation of the TV region is particularly challenging, even for expert raters, because it is difficult to distinguish edema from non-enhancing tumor. This can also be seen from the large spread for the TV region in figure 4. Observer independent sub-compartmental volumetry may identify new research areas where structural and dynamic texture parameters can be defined for the different tumor sub-compartments and may lead to new theories of tumor spread or therapy response by integrating histopathological features within the TV+ regions [45], [46].

Limitations of this study include the fact that our analysis is restricted to pre-surgical tumor volumetry. Future work should address the accuracy of longitudinal volumetric measures and evaluate whether complementary ADC and perfusion imaging may improve the clinical yield of “tailored” multi-parameter analysis.

## Conclusions

In summary, we demonstrated that BraTumIA supports radiologists and clinicians with accurate measures of cross sectional 2-dimensional tumor extensions that are equivalent to manual tumor delineation, and provides CETV tumor volumes that have lower variability than manual ratings.

## Supporting Information

Data S1 **This file includes Method S1 and Tables S1 to S6.** Method S1: Details of the segmentation algorithm. Table S1: Automated versus manual segmentation of expert 1. Table S2: Automated versus manual segmentation of expert 2. Table S3: Inter-observer agreement of manual segmentations of expert 1 and expert 2. Table S4: Statistical analysis (Wilcoxon signed rank test) of agreement between automatic and manual segmentations. Table S5: Spearman rank correlation coefficients of automatic and manual segmentations. Table S6: Kappa coefficient of mutual agreement between different automatic and manual segmentations in all tumor sub-compartments.
(PDF)

## References

[1] Stupp R, Mason WP, van den Bent MJ, Weller M, Fisher B, et al (2005) Radiotherapy plus concomitant and adjuvant temozolomide for glioblastoma. N Engl J Med 352: 987–996 10.1056/NEJMoa043330 15758009

[2] Stupp R, Hegi ME, Mason WP, van den Bent MJ, Taphoorn MJB, et al (2009) Effects of radiotherapy with concomitant and adjuvant temozolomide versus radiotherapy alone on survival in glioblastoma in a randomised phase III study: 5-year analysis of the EORTC-NCIC trial. Lancet Oncol 10: 459–466 10.1016/S1470-2045(09)70025-7 19269895

[3] Stummer W, Reulen H-J, Meinel T, Pichlmeier U, Schumacher W, et al (2008) Extent of resection and survival in glioblastoma multiforme: identification of and adjustment for bias. Neurosurgery 62: 564–76 discussion 564–76 10.1227/01.neu.0000317304.31579.17 18425006

[4] Seidel K, Beck J, Stieglitz L, Schucht P, Raabe A (2013) The warning-sign hierarchy between quantitative subcortical motor mapping and continuous motor evoked potential monitoring during resection of supratentorial brain tumors. J Neurosurg 118: 287–296 10.3171/2012.10.JNS12895 23198802

[5] Seidel K, Beck J, Stieglitz L, Schucht P, Raabe A (2012) Low-threshold monopolar motor mapping for resection of primary motor cortex tumors. Neurosurgery 71: 104–14 discussion 114–5 10.1227/NEU.0b013e31824c02a0 22270233

[6] Devaux BC, O'Fallon JR, Kelly PJ (1993) Resection, biopsy, and survival in malignant glial neoplasms. A retrospective study of clinical parameters, therapy, and outcome. J Neurosurg 78: 767–775 10.3171/jns.1993.78.5.0767 8468607

[7] Sanai N, Berger MS (2011) Extent of resection influences outcomes for patients with gliomas. Rev Neurol (Paris) 167: 648–654 10.1016/j.neurol.2011.07.004 21903234

[8] Hammoud MA, Sawaya R, Shi W, Thall PF, Leeds NE (1996) Prognostic significance of preoperative MRI scans in glioblastoma multiforme. J Neurooncol 27: 65–73. 8699228 10.1007/BF00146086

[9] Lacroix M, Abi-Said D, Fourney DR, Gokaslan ZL, Shi W, et al (2001) A multivariate analysis of 416 patients with glioblastoma multiforme: prognosis, extent of resection, and survival. J Neurosurg 95: 190–198 10.3171/jns.2001.95.2.0190 11780887

[10] Weltens C, Menten J, Feron M, Bellon E, Demaerel P, et al (2001) Interobserver variations in gross tumor volume delineation of brain tumors on computed tomography and impact of magnetic resonance imaging. Radiother Oncol 60: 49–59. 11410304 10.1016/s0167-8140(01)00371-1

[11] Mazzara GP, Velthuizen RP, Pearlman JL, Greenberg HM, Wagner H (2004) Brain tumor target volume determination for radiation treatment planning through automated MRI segmentation. Int J Radiat Oncol Biol Phys 59: 300–312 10.1016/j.ijrobp.2004.01.026 15093927

[12] Deeley MA, Chen A, Datteri R, Noble JH, Cmelak AJ, et al (2011) Comparison of manual and automatic segmentation methods for brain structures in the presence of space-occupying lesions: a multi-expert study. Phys Med Biol 56: 4557–4577 10.1088/0031-9155/56/14/021 21725140 PMC3153124

[13] Deeley MA, Chen A, Noble J, Cmelak A, Donnelly E, et al (2013) Segmentation editing improves efficiency while reducing inter-expert variation and maintaining accuracy for normal brain tissues in the presence of space-occupying lesions. Phys Med Biol 58: 4071–4097 10.1088/0031-9155/58/12/4071 23685866 PMC3744837

[14] Bauer S, Wiest R, Nolte L-P, Reyes M (2013) A survey of MRI-based medical image analysis for brain tumor studies. Phys Med Biol 58: R97–R129 10.1088/0031-9155/58/13/R97 23743802

[15] Corso JJ, Sharon E, Dube S, El-Saden S, Sinha U, et al (2008) Efficient multilevel brain tumor segmentation with integrated bayesian model classification. IEEE Trans Med Imaging 27: 629–640 10.1109/TMI.2007.912817 18450536

[16] Verma R, Zacharaki EI, Ou Y, Cai H, Chawla S, et al (2008) Multiparametric tissue characterization of brain neoplasms and their recurrence using pattern classification of MR images. Acad Radiol 15: 966–977 10.1016/j.acra.2008.01.029 18620117 PMC2596598

[17] Bauer S, Nolte L-P, Reyes M (2011) Fully automatic segmentation of brain tumor images using support vector machine classification in combination with hierarchical conditional random field regularization. In: Fichtinger G, Martel A, Peters T, editors. MICCAI. International Conference on Medical Image Computing and Computer-Assisted Intervention. Lecture Notes in Computer Science. Toronto: Springer Berlin Heidelberg, Vol. 14. pp. 354–361. doi:10.1007/978-3-642-23626-6_44. 10.1007/978-3-642-23626-6_44 22003719

[18] Menze BH, Van Leemput K, Lashkari D, Weber M-A, Ayache N, et al (2010) A generative model for brain tumor segmentation in multi-modal images. Med Image Comput Comput Assist Interv 13: 151–159. 10.1007/978-3-642-15745-5_19 PMC3050038 20879310

[19] Zikic D, Glocker B, Konukoglu E, Criminisi A, Demiralp C, et al. (2012) Decision Forests for Tissue-specific Segmentation of High-grade Gliomas in Multi-channel MR. MICCAI - Medical Image Computing and Computer Assisted Interventions. Nice: Springer LNCS, Vol. m. pp. 1–8. 10.1007/978-3-642-33454-2_46 23286152

[20] Menze B, Jakab A, Bauer S, Kalpathy-Cramer J, Farahani K, et al. (n.d.) The Multimodal Brain Tumor Image Segmentation Benchmark (BRATS). 10.1109/TMI.2014.2377694 PMC4833122 25494501

[21] Wen PY, Macdonald DR, Reardon D a, Cloughesy TF, Sorensen a G, et al (2010) Updated response assessment criteria for high-grade gliomas: response assessment in neuro-oncology working group. J Clin Oncol 28: 1963–1972 10.1200/JCO.2009.26.3541 20231676

[22] Gooya A, Pohl KM, Bilello M, Cirillo L, Biros G, et al (2012) GLISTR: glioma image segmentation and registration. IEEE Trans Med Imaging 31: 1941–1954 10.1109/TMI.2012.2210558 22907965 PMC4371551

[23] Tustison N, Wintermark M, Durst C, Avants B (2013) ANTs and _Arboles. MICCAI BraTS Workshop. Nagoya: Miccai Society.

[24] Miller AB, Hoogstraten B, Staquet M, Winkler A (1981) Reporting results of cancer treatment. Cancer 47: 207–214. 7459811 10.1002/1097-0142(19810101)47:1<207::aid-cncr2820470134>3.0.co;2-6

[25] Gállego Pérez-Larraya J, Lahutte M, Petrirena G, Reyes-Botero G, González-Aguilar A, et al (2012) Response assessment in recurrent glioblastoma treated with irinotecan-bevacizumab: comparative analysis of the Macdonald, RECIST, RANO, and RECIST + F criteria. Neuro Oncol 14: 667–673 10.1093/neuonc/nos070 22492961 PMC3337315

[26] Fedorov A, Beichel R, Kalpathy-Cramer J, Finet J, Fillion-Robin J-C, et al (2012) 3D Slicer as an image computing platform for the Quantitative Imaging Network. Magn Reson Imaging 30: 1323–1341 10.1016/j.mri.2012.05.001 22770690 PMC3466397

[27] Gutman D a, Cooper L a D, Hwang SN, Holder C a, Gao J, et al. (2013) MR Imaging Predictors of Molecular Profile and Survival: Multi-institutional Study of the TCGA Glioblastoma Data Set. Radiology: 1–10. doi:10.1148/radiol.13120118. 10.1148/radiol.13120118 PMC3632807 23392431

[28] Bauer S, Fejes T, Reyes M (2012) A Skull-Stripping Filter for ITK.

[29] Ibanez L, Schroeder W, Ng L, Cates J, others (2005) The ITK software guide. 2nd ed. Kitware.

[30] Bauer S, Fejes T, Slotboom J, Wiest R, Nolte L-P, et al. (2012) Segmentation of Brain Tumor Images Based on Integrated Hierarchical Classification and Regularization. In: Menze B, Jakab A, Bauer S, Reyes M, Prastawa M, et al.., editors. MICCAI BraTS Workshop. Nice: Miccai Society.

[31] Criminisi A, Shotton J, editors (2013) Decision Forests for Computer Vision and Medical Image Analysis. London: Springer London. doi:10.1007/978-1-4471-4929-3.

[32] Crum WR, Camara O, Hill DLG (2006) Generalized overlap measures for evaluation and validation in medical image analysis. IEEE Trans Med Imaging 25: 1451–1461 10.1109/TMI.2006.880587 17117774

[33] Altman D, Bland J (1994) Diagnostic tests 2: predictive values. BMJ Br Med J 309: 16104. 10.1136/bmj.309.6947.102 PMC2540558 8038641

[34] Altman D, Bland J (1994) Diagnostic tests 1: sensitivity and specificity. BMJ Br Med J. doi:10.1136/bmj.308.6943. 10.1136/bmj.308.6943.1552 PMC2540489 8019315

[35] Wilcoxon F (1945) Individual comparisons by ranking methods. Biometrics Bull 1: 80–83.

[36] Spearman C (1904) The proof and measurement of association between two things. Am J Psychol 15: 72–101. 3322052

[37] Friedman M (1952) The Use of Ranks to Avoid the Assumption of Normality Implicit in the Analysis of Variance. J Am Stat Assoc 32: 675–701.

[38] Kruskal WH, Wallis WA (1952) Use of Ranks in One-Criterion Variance Analysis. J Am Stat Assoc 47: 583–621 10.2307/2280779

[39] Macdonald DR, Cascino TL, Schold SC, Cairncross JG (1990) Response criteria for phase II studies of supratentorial malignant glioma. J Clin Oncol 8: 1277–1280. 2358840 10.1200/JCO.1990.8.7.1277

[40] Reuter M, Gerstner ER, Rapalino O, Batchelor TT, Rosen B, et al. (2014) Impact of MRI head placement on glioma response assessment. J Neurooncol. doi:10.1007/s11060-014-1403-8. 10.1007/s11060-014-1403-8 PMC4026260 24566765

[41] Egger J, Kapur T, Fedorov A, Pieper S, Miller J V, et al (2013) GBM Volumetry using the 3D Slicer Medical Image Computing Platform. Sci Rep 3: 1364 10.1038/srep01364 23455483 PMC3586703

[42] Heckel F, Moltz JH, Tietjen C, Hahn HK (2013) Sketch-Based Editing Tools for Tumour Segmentation in 3D Medical Images. Comput Graph Forum 32: 144–157 10.1111/cgf.12193

[43] Ellingson BM, Cloughesy TF, Zaw T, Lai A, Nghiemphu PL, et al (2012) Functional diffusion maps (fDMs) evaluated before and after radiochemotherapy predict progression-free and overall survival in newly diagnosed glioblastoma. Neuro Oncol 14: 333–343 10.1093/neuonc/nor220 22270220 PMC3280805

[44] Brandsma D, van den Bent MJ (2009) Pseudoprogression and pseudoresponse in the treatment of gliomas. Curr Opin Neurol 22: 633–638 10.1097/WCO.0b013e328332363e 19770760

[45] Slotboom J, Schaer R, Ozdoba C, Reinert M, Vajtai I, et al (2008) A novel method for analyzing DSCE-images with an application to tumor grading. Invest Radiol 43: 843–853 10.1097/RLI.0b013e3181893605 19002056

[46] Schucht P, Ghareeb F, Duffau H (2013) Surgery for low-grade glioma infiltrating the central cerebral region: location as a predictive factor for neurological deficit, epileptological outcome, and quality of life. J Neurosurg 119: 318–323 10.3171/2013.5.JNS122235 23767891

