# Supplementary material for: Multi-Modal Glioblastoma Segmentation: Man versus Machine
Source: PLoS One. 2014 May 7;9(5):e96873. doi: 10.1371/journal.pone.0096873 (PMC4013039; doi:10.1371/journal.pone.0096873)
Supplement: Data S1 — This file includes Method S1 and Tables S1 to S6. Method S1: Details of the segmentation algorithm. Table S1: Automated versus manual segmentation of expert 1. Table S2: Automated versus manual segmentation of expert 2. Table S3: Inter-observer agreement of manual segmentations of expert 1 and expert 2. Table S4: Statistical analysis (Wilcoxon signed rank test) of agreement between automatic and manual segmentations. Table S5: Spearman rank correlation coefficients of automatic and manual segmentations. Table S6: Kappa coefficient of mutual agreement between different automatic and manual segmentations in all tumor sub-compartments. (PDF) [file pone.0096873.s001.pdf]

SUPPLEMENTARY MATERIAL

Method S1: Details of the segmentation algorithm

We employ a fully automatic method for brain tumor segmentation, which is based on classification with integrated hierarchical regularization. It subcategorizes healthy tissues into CSF, WM, GM and pathologic tissues into necrotic, active, non-enhancing and edema compartments.

After pre-processing (denoising, bias-field correction, rescaling and histogram matching), the segmentation task is modeled as an energy minimization problem in a conditional random field (CRF) formulation. The energy consists of the sum of the singleton potentials in the first term and the pairwise potentials in the second term of equation (1). The expression is minimized using linear programming strategies in a hierarchical way.

$$E = \sum_i V(y_i, \mathrm{x}_i) + \sum_{ij} W(y_i, y_j, \mathrm{x}_i, \mathrm{x}_j) \quad (1)$$

The singleton potentials $V(y_i, \mathrm{x}_i)$ are computed according to equation (2), where $\tilde{y}_i$ is the label output from a classifier, x_i is the feature vector and δ is the Kronecker-δ function.

$$V(y_i, \mathrm{x}_i) = p(\tilde{y}_i|\mathrm{x_i}) \cdot (1 - \delta(\tilde{y}_i, y_i)) \quad (2)$$

We use a decision forest as a classifier, which has the advantage of being able to handle multi-class problems and providing a probabilistic output. The probabilistic output is used for the weighting factor $p(\tilde{y}_i|\mathrm{x}_i)$ in equation (2), in order to control the degree of spatial regularization. A 256-dimensional feature vector is used for the classifier, which combines the intensities in each modality with the first-order textures (mean, variance, skewness, kurtosis, energy, entropy) computed from local patches, statistics of intensity gradients in a local neighborhood and symmetry features across the mid-sagittal plane and location features. The pairwise potentials $W(y_i, y_j, \mathrm{x}_i, \mathrm{x}_j)$ account for the spatial regularization. In equation (3) $w_s(i,j)$ is a weighting function, which depends on the voxel spacing in each dimension. The term $(1 - \delta(y_i, y_j))$ penalizes different labels of adjacent voxels, while the intensity term $\exp\left(\frac{-\mathrm{PCD}(\mathrm{x}_i - \mathrm{x}_j)}{2 \cdot \bar{x}}\right)$ regulates the degree of smoothing based on the local intensity variation, where PCD is a pseudo-Chebyshev distance and $\bar{x}$ is a generalized mean intensity. $D(y_i, y_j)$ allows us to incorporate prior knowledge by penalizing different tissue adjacencies individually.

$$W(y_i, y_j, \mathrm{x}_i, \mathrm{x}_j) = w_s(i,j) \cdot (1 - \delta(y_i, y_j)) \cdot \exp\left(\frac{-\mathrm{PCD}(\mathrm{x}_i - \mathrm{x}_j)}{2 \cdot \bar{x}}\right) \cdot D(y_i, y_j) \quad (3)$$

Table S1: Comparison of automated versus manual segmentation of expert 1 (AE1)

	**Dice**	**PPV**	**Sensitivity**	**relVolErr**	**absVolErr**
**TV+**					
median	0.84	0.80	0.88	0.20	21.09
mean	0.80	0.76	0.87	0.27	20.39
stddev	0.12	0.17	0.07	0.32	16.27
Lower 95%	0.75	0.68	0.84	0.14	13.68
Upper 95%	0.85	0.83	0.89	0.40	27.12
**TV**					
median	0.70	0.76	0.74	0.29	11.03
mean	0.66	0.69	0.70	0.40	14.45
stddev	0.14	0.18	0.22	0.44	14.28
Lower 95%	0.60	0.61	0.60	0.21	8.55
Upper 95%	0.72	0.76	0.78	0.60	20.35
**CETV**					
median	0.66	0.62	0.78	0.39	6.67
mean	0.63	0.61	0.73	0.57	7.21
stddev	0.12	0.15	0.22	0.55	6.01
Lower 95%	0.58	0.55	0.64	0.34	4.73
Upper 95%	0.68	0.67	0.82	0.80	9.69

AE1: Automatic Segmentation versus Manual Segmentation of Expert rater 1 (ER1); PPV = Positive predictive Values; RelVolErr= relative volume error, AbsVolErr= absolute volume error (cm^3); TV= complete tumor volume (encompassing the enhancing part of the tumor, the non-enhancing part of the tumor and the necrotic core); TV+ = TV plus edema; CETV = contrast enhancing tumor volume (CETV)

Table S2: Comparison of automated versus manual segmentation of expert 2 (AE2)

	**Dice**	**PPV**	**Sensitivity**	**relVolErr**	**absVolErr**
**TV+**					
median	0.81	0.74	0.88	0.26	18.16
mean	0.77	0.70	0.88	0.40	25.45
stddev	0.13	0.18	0.07	0.48	17.26
Lower 95%	0.71	0.62	0.84	0.19	18.32
Upper 95%	0.82	0.77	0.91	0.60	32.58
**TV**					
median	0.71	0.73	0.72	0.28	9.65
mean	0.66	0.69	0.69	0.40	13.39
stddev	0.13	0.17	0.18	0.47	14.44
Lower 95%	0.60	0.62	0.61	0.20	7.43
Upper 95%	0.71	0.77	0.76	0.60	19.35
**CETV**					
median	0.55	0.43	0.83	0.69	6.24
mean	0.53	0.43	0.79	1.32	9.42
stddev	0.16	0.16	0.13	1.61	8.23
Lower 95%	0.47	0.36	0.73	0.66	6.03
Upper 95%	0.60	0.50	0.84	1.99	12.82

AE2: Automatic Segmentation versus Manual Segmentation of Expert rater 2 (ER2); PPV = Positive predictive Values; RelVolErr= relative volume error, AbsVolErr= absolute volume error (cm^3); TV= complete tumor volume (encompassing the enhancing part of the tumor, the non-enhancing part of the tumor and the necrotic core); TV+ = TV plus edema; CETV = contrast enhancing tumor volume

Table S3: Inter-observer comparison of manual segmentation of expert 1 and manual segmentation of expert 2 (IR)

	**Dice**	**PPV**	**Sensitivity**	**relVolErr**	**absVolErr**
**TV+**					
median	0.86	0.92	0.82	0.16	13.70
mean	0.85	0.89	0.82	0.16	17.77
stddev	0.05	0.09	0.08	0.09	15.98
Lower 95%	0.82	0.85	0.78	0.12	11.17
Upper 95%	0.87	0.93	0.85	0.19	24.36
**TV**					
median	0.78	0.75	0.80	0.19	4.63
mean	0.74	0.75	0.77	0.23	7.82
stddev	0.13	0.12	0.17	0.21	7.2
Lower 95%	0.69	0.70	0.70	0.14	4.84
Upper 95%	0.80	0.80	0.84	0.31	10.80
**CETV**					
median	0.55	0.79	0.51	0.42	4.56
mean	0.57	0.78	0.52	0.45	8.44
stddev	0.16	0.13	0.24	0.22	10.79
Lower 95%	0.50	0.72	0.41	0.36	3.40
Upper 95%	0.63	0.83	0.61	0.54	12.90

IR: Manual Segmentation of ER1 (Expert Rater 1) versus Manual Segmentation of ER2 (Expert Rater 2); PPV = Positive predictive Values; RelVolErr= relative volume error, AbsVolErr= absolute volume error (cm^3); TV= complete tumor volume (encompassing the enhancing part of the tumor, the non-enhancing part of the tumor and the necrotic core); TV+ = TV plus edema; CETV = contrast enhancing tumor volume

Table S4: Wilcoxon signed ranks test
Automatic segmentation versus manual segmentation of expert 1 (AE1) compared to manual inter-observer segmentation (IR) and automatic segmentation versus manual segmentation of expert 2 (AE2) compared to manual inter-observer segmentation (IR)

Dice coefficient	AE1 -IR	AE2- IR
	p	p
TV +	0.01	0.00
TV	0.00	0.00
CETV	0.21	0.64

PPV	AE1 -IR	AE2- IR
	p	p
TV +	0.00	0.00
TV	0.08	0.13
CETV	0.00	0.00

Sensitivity	AE1 -IR	AE2- IR
	p	p
TV +	0.45	0.35
TV	0.09	0.03
CETV	0.00	0.00

absVolErr	AE1 -IR	AE2- IR
	p	p
TV +	0.57	0.32
TV	0.14	0.28
CETV	0.98	0.30

relVolErr	AE1 -IR	AE2- IR
	p	p
TV +	0.16	0.01
TV	0.32	0.85
CETV	0.42	0.30

PPV = Positive predictive Values; RelVolErr= relative volume error, AbsVolErr= absolute volume error (cm^3); TV= complete tumor volume (encompassing the enhancing part of the tumor, the non-enhancing part of the tumor and the necrotic core); TV+ = TV plus edema; CETV = contrast enhancing tumor volume

Table S5: Spearmans rank correlation coefficients (ρ) of TV+, TV and CETV
Automatic segmentation versus manual segmentation of expert 1 (AE1) compared to automatic versus manual segmentation of expert 2 (AE2); automatic segmentation versus manual segmentation of expert 1 (AE1) compared to manual inter-observer segmentation (IR); and automatic segmentation versus manual segmentation of expert 2 (AE2) compared to manual inter-observer segmentation (IR)

Dice coefficient	AE1 versus AE2			AE1 versus IR			AE2 versus IR		
	**ρ**	p	Lower 95%- Upper 95% CI	**ρ**	p	Lower 95%- Upper 95% CI	**ρ**	p	Lower 95%- Upper 95% CI
TV +	0.85	0.00	0.70 - 0.93	0.74	0.00	0.50 - 0.88	0.79	0.00	0.51 - 0.92
TV	0.90	0.00	0.73 - 0.96	0.68	0.00	0.43 - 0.82	0.75	0.00	0.54 - 0.85
CETV	0.42	0.03	0.02 - 0.73	0.51	0.00	0.14 - 0.76	0.84	0.00	0.57 - 0.94

Spearmann's rank correlation coefficients (ρ); TV= complete tumor volume (encompassing the enhancing part of the tumor, the non-enhancing part of the tumor and the necrotic core); TV+ = TV plus edema; CETV = contrast enhancing tumor volume; Lower 95% and Upper 95% CI (confidence interval)

Table S6: Kappa coefficient for mutual agreement between different raters in all tumor sub-compartments, evaluated on the 25 study patients (background and healthy tissues excluded).

	**AE1**	**AE2**	**IR**
**Median**	0.37	0.32	0.46
**Mean**	0.41	0.36	0.46
**Stddev**	0.15	0.14	0.11

Automatic segmentation versus manual segmentation of expert 1 (AE1), automatic segmentation versus manual segmentation of expert 2 (AE2) and manual inter-observer segmentation (IR)
